# The Records of Stannington Children’s Sanatorium: Charting Half a Century of Tuberculosis Care

**DOI:** 10.1093/shm/hkw027

**Published:** 2016-04-14

**Authors:** Karen Rushton

**Keywords:** tuberculosis, archives, sanatoria

## Abstract

This article explores the historic records of Stannington Children’s Tuberculosis Sanatorium focusing largely on the 5,041 patient records and 14,660 radiographs that make up the bulk of the collection and span from the 1930s to the 1960s. By taking a handful of illustrations from within the collection, it aims to demonstrate the various avenues of research available as well as the unique nature of the collection owing to its focus on children, with the comprehensive nature of its records making it invaluable. The sanatorium’s records are made particularly pertinent by the fact that they span the pre- to the post-antibiotic era charting changes in treatment as well as offering details on a range of other issues such as social background and stigma.

## Introduction

The records of Stannington Sanatorium, the UK’s first purpose-built children’s tuberculosis sanatorium, were retrieved by Northumberland Archives upon its closure in 1984. Nevertheless, it is only in the past year that any serious attempts at cataloguing the collection have been possible thanks to funding from the Wellcome Trust. As a result, the wealth of records contained within the collection will be readily available for future research.

The case histories of numerous former Stannington patients were used to inform the research of Drs Miller, Seal, and Taylor in their seminal work on tuberculosis in children arising from investigations in the Northeast of England in the 1950s and 1960s.^1^ Since this time the records have remained largely untouched with the exception of a 2003 PhD thesis offering a demographic analysis of Stannington patients but no exploration at the individual level.^2^ As of September 2015 another student has begun a PhD utilising the radiographs from the collection in assessing their suitability as a comparative tool against the archaeological record.

## What was the Significance of Stannington Sanatorium?

Under the auspices of a local charity, The Poor Children’s Holiday Association, Stannington Sanatorium officially opened in 1907 in Northumberland near to the village of Stannington. Thanks to the efforts of a few individuals determined to tackle the problem of childhood poverty in the area and in turn the scourge of tuberculosis, private funding and donations were secured to purchase White House Farm at Stannington. The site was used to first build a farm colony for the training of rescued street boys and the construction of the sanatorium soon followed. The charity had been running day trips and later longer holidays to the seaside for impoverished children from the Newcastle and Gateshead area for several years up to this point but, owing to their medical condition, tuberculous children were often excluded from this and the establishment of the sanatorium allowed these children to benefit from the charity’s efforts.

From its opening in 1907 the sanatorium continued to dedicate all its time and effort solely to treating tuberculous children up to 1953 whereupon it became a general children’s hospital before closing completely in 1984. During its 46 years of treatment of tuberculosis, the institution treated thousands of children and was witness to many advances in medical treatments and changes in social care and public health schemes all evidenced through its records.

The whole country saw consistently high death rates from tuberculosis throughout the first half of the twentieth century and Medical Officer of Health reports from this period indicate that the counties of Northumberland and Durham often had some of the highest rates.^3^ However, statistical data taken from the records of Stannington Sanatorium show it to have had particularly low death rates in comparison. For example, in 1942 there was a death rate of 46.38 per cent in Northumberland against 3.3 per cent in the sanatorium.^4^ There would have been many factors influencing this statistic, not least the recognised resilience of children in comparison to adults.^5^ In addition we see patients discharged from Stannington described as having ‘no medical improvement’ (NMI) and who are essentially being sent home to die. However, even with these patients are accounted for, the death/NMI rate remains very low and it is something that potentially warrants further investigation.

Nicely summarised by T. Waldron: ‘tuberculosis is *par excellence* a disease of poverty, overcrowding and malnutrition’.^6^ The incidence of tuberculosis in the western world nowadays is very low and is not seen by most to be a pertinent threat with many of the underlying factors having been tackled and effective public health measures in place. Nonetheless, tuberculosis continues to be prevalent across the developing world, being the greatest killer caused by a single infectious agent after HIV/AIDs.^7^ Whilst the medical histories of the children found in Stannington Sanatorium may appear to bear no relevance to the experience of children growing up in the UK today, the same cannot be said for large swathes of the global population. Even with this borne in mind it is still important to note that tuberculosis in the UK is far from being eradicated completely. Public Health England’s 2013 report on tuberculosis highlights the prevalence of the disease in the UK in comparison to other western European countries, with the majority of cases breaking out amongst the non UK-born population coming from countries where tuberculosis is still widespread. The report also highlights the continuing prevalence of the disease in urban centres and amongst those with social risk factors for tuberculosis.^8^ It would seem the topic of tuberculosis, encompassing its spread, effective treatments and the demographics of the disease, is still a relevant one with the records of Stannington Sanatorium having much to offer in tracking the history of the development of tuberculosis treatment.

In this article I will first address those key series of records within the collection that are common to most hospital collections; secondly I will look to the more distinctive records related to Stannington that make it stand out as a collection of particular interest, and finally I will discuss the practicalities surrounding access to the records.

## Core of the Collection

The collection consists of 120 linear feet of records comprising administrative, financial, staffing and patient files and also including one linear foot of photographic material. However, the bulk of the records and arguably the highlight, are the 5,041 case files and 14,660 corresponding patient radiographs. Altogether there are 100 linear feet of patient records, the contents of which include data on other family members, living conditions, medical history, temperature charts, X-ray reports, pathology reports, details of progress, treatments administered and any correspondence with family members or local authorities. The earliest case files date from the late 1930s with a near complete run up until 1966, by which point the sanatorium was a general children’s hospital.

There are only very occasional gaps in the run of patient records, and the sanatorium’s incorporation into the NHS in 1948 probably helped to ensure their long-term survival. Their comprehensiveness makes them an ideal resource for demographic analyses as well as charting developments in the most commonly provided treatments and the assessment of their efficacy. The period from the late 1930s to 1966 saw a great number of developments in tuberculosis care with the introduction of effective antibiotics in 1947, implementation of public health schemes, and the eventual decline of the disease in the 1960s, all of which can be witnessed in some form through the patient case files. The patient records also lend themselves to studies of some of the social issues commonly associated with tuberculosis, such as living conditions and the stigma attached to the disease.

The following letter dated May 1955 and extracted from one young boy’s file encapsulates just how the stigma of TB could affect a patient’s life long after they were deemed to be cured and also illustrates the rich resource of the correspondence held in patient files:

… After his first check-up, Dr Rowlands was very pleased with the result and his second proved satisfactory too except I came home troubled in mind about what the doctor said. It wasn’t Dr Rowlands I saw.

I asked if [38/1953] could go swimming and was told in no uncertain terms that I should consider the feelings of other mothers. Would I like my child to swim with one so recently treated for T.B? He pointed out that he had people on his books who had been negative for years but who couldn’t get employment because people objected to working beside them. Also that we never use the word ‘cured’ in regard to TB giving a list of words that were used, not one of them meaning cured.

[38/1953] listened intently and I saw all the good work done at the hospital, being thrown away in a few minutes. …^9^

This particular instance seems to hold more power than other examples simply because the prejudice is being upheld by a member of the medical community, who we might otherwise expect to want to see the destruction of such views, often counterproductive to treatment and prevention. Items of correspondence held within patient files are a particularly rich source of information when investigating any lasting stigma that tuberculosis may have had. The same can also be said for information on other social issues such as living conditions and housing.

The clinical work carried out by the sanatorium is further illustrated by a series of different treatment registers held within the collection, some of which pre-date the extant patient records helping us to chart the different practices employed over a longer period. The treatment registers include a register of artificial pneumothorax cases, an operations register, X-ray registers and a register of splints issued for the treatment of patients with tuberculosis of the bones and joints. The register of artificial pneumothorax cases, for example, extends from 1922 to 1936 and for each patient gives details of the diagnosis, when treatment began, how much air was inserted and how often, any side effects and indications of success or failure.

In addition to the clinical records, several series of administrative, financial and staffing records exist, most of which are in keeping with the general infrastructure of any hospital. These include annual reports, committee minutes and staff registers, and at first glance can easily be neglected in favour of the more obviously richly detail clinical records, but their value should not be so readily overlooked. The development of the sanatorium and the reasoning behind its general operation and many of the medical treatments offered, as well as its place in the wider national battle against TB, can often be more easily discerned from these groups of records than from patient files and clinical registers. Amongst the more standard administrative records can be found slightly less commonplace items such as a publicity brochure issued in 1936 showcasing their facilities and success stories.

**Fig. 1.**
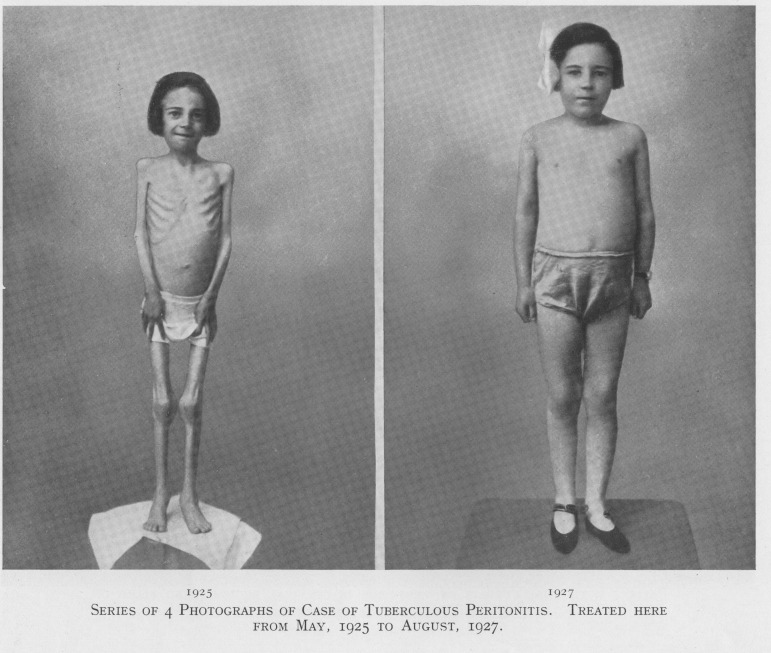
*Source*: Northumberland Archives: HOSP/STAN/9/1/1.

Figure 1 is one page from this brochure and, whilst the case in question predates the extant patient files, the case was reported at the time by the Medical Superintendent Dr T.C. Hunter in *The Lancet*; a good example of how contemporary published sources can often support the archival record, although the lack of representativeness of published cases must be considered. The girl in question was admitted to the sanatorium with peritoneal tuberculosis on 1 May 1925 when she was aged 8 years and 11 months, with a gap of just over two years between the two photographs being taken. Hunter gives us a detailed report of her case and treatment with heliotherapy, the main approach for the treatment of the children admitted to Stannington where surgery was not an option and before any drug therapies had been developed. Here we get a rare detailed and illustrated report of the treatment of TB with heliotherapy.

Hunter’s report tells us that upon her admission she was 3 ft 8¾ in tall and weighed only 2st 2lb 4oz. Artificial sunlight treatment was instituted immediately through the use of a mercury-vapour lamp for two minutes each day, rising to five minutes. However, her condition in the first few months continued to worsen and she lost an additional 3¼ lb. and a decision was made to stop the artificial sunlight treatment. During the spring of 1926 it was possible for natural sunlight treatment to commence and at the patient’s request artificial sunlight treatment was recommenced too. Her condition then began to improve and following the institution of treatment by collosol iodine in addition to the heliotherapy Hunter describes her progress from then on as remarkable.^10^

The main principle behind heliotherapy in combination with good food and rest was of strengthening the patient and so enabling them to better resist and fight off the disease, an idea popularised early on by Dr Auguste Rollier.^11^ Writing in the *British Medical Journal* Dr Allison, Stannington Sanatorium’s first visiting physician and one of the key architects of its establishment, expressly supports the ideas laid down by Rollier and these can be said to form the basis of Stannington’s early work.^12^ It is possible to investigate the efficacy of these sorts of measures through the early case files of patients where few other proactive measures were undertaken in the context of the extent, type and progression of the disease in each.

## What Makes Stannington Unique?

Archives of other tuberculosis hospitals exist across the country including collections at The Royal London Hospital Archives, Lothian Health Services Archives and of the Papworth Village Settlement held by Cambridgeshire Archives, all undoubtedly rich with information for the study of the history of tuberculosis. Nevertheless the Stannington Collection remains unique in part for its focus on children but also owing to the presence of thousands of radiographic images within the collection. The radiographs are not unique to Stannington Sanatorium in the sense that they were the only place to use them, as this was not true—they were a common diagnostic tool at most sanatoria during this period—but their uniqueness to the collection stems simply from the fact that they have survived.

The 14,660 radiographs complement the files illustrating the progression of tuberculosis in each patient, the course of different treatments that were administered and also other medical ailments either unrelated to tuberculosis or occurring as a consequence. Spanning the years 1936–1955 the radiographs are grouped by patient relating to 2,243 individual patients in total. Owing to concerns about the stability of the radiographs the majority of them were transferred to microfiche and the originals destroyed in 1988, however the archive does still contain 326 of the original radiographs. Following the installation of an X-ray plant in the sanatorium in 1920, all patients would have been routinely X-rayed throughout their stay as a matter of course.^13^ As such the 14,660 radiographs include ample examples of the different manifestations of tuberculosis, covering pulmonary cases, tuberculosis of the bones and joints and other extra-pulmonary forms of the disease.

Exploration of the treatment of the most common manifestation of the disease, pulmonary tuberculosis, in one particular patient nicely encapsulates some of the key surgical procedures employed at Stannington Sanatorium and how the manifestation of the disease and the effect of treatment can be readily illustrated by the radiographs. Prior to the introduction of antibiotics, surgical procedures were the only proactive ways of tackling the disease available to the medical community. Even following the advent of antibiotics, surgical treatments were in use for many years with a variety of procedures in use for the treatment of the different types of tuberculosis.

Patient 95/1947, a 12-year-old female, was admitted to the sanatorium on 4 September 1947 presenting with poor appetite and weight loss weighing only 4st 0lb 6oz. She had no cough and no other physical symptoms were reported. Radiographic evidence obtained following her admission supports the diagnosis of pulmonary tuberculosis. The report made on her first X-ray reads as follows, ‘Tuberculous infiltration of both upper lobes with a large cavity in the mid-zone & a smaller one at the left apex. There are several small calcified foci in the right upper lobe.’^14^

**Fig. 2.**
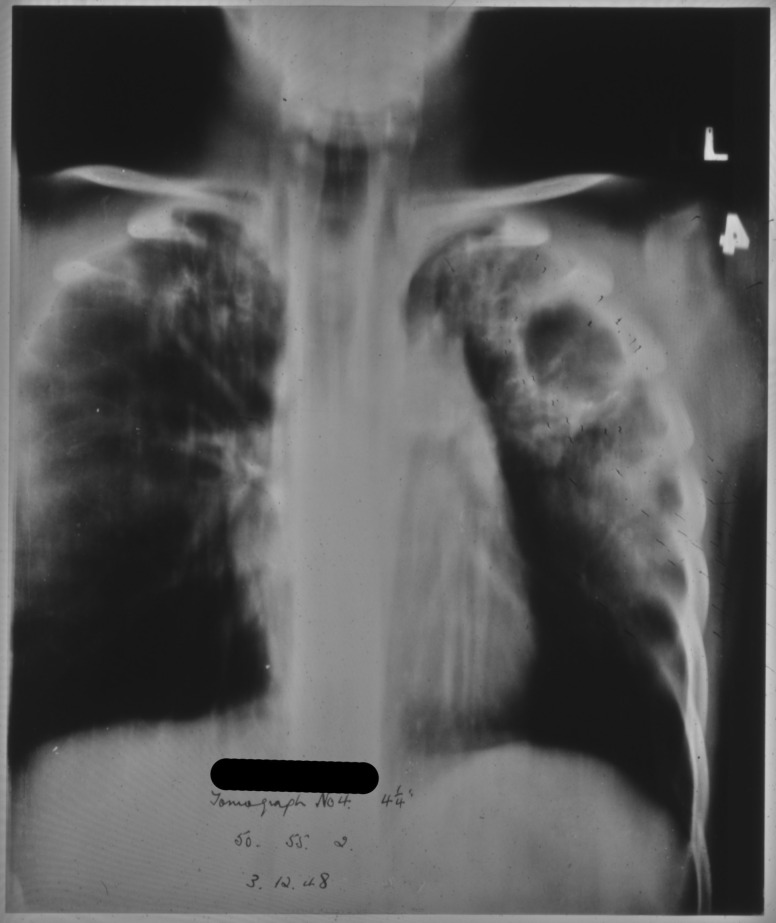
Tomograph of 12-year-old female showing large cavity in the left lung. *Source*: Northumberland Archives: HOSP/STAN/7/1/2/1444_19.

This child’s doctor initially described her outlook as being very poor and did not recommend any particular course of treatment. During the first few months of her stay additional X-rays showed great improvements in the condition of her right lung but a continued deterioration in the left lung was observed for many months afterwards, as seen in Figure 2, a tomograph taken in December 1948. Even after her doctor’s initial pessimistic evaluation, attempts at treatment did begin in January 1948 starting with an attempted artificial pneumothorax of the left lung.

Artificial pneumothorax treatment aimed to rest the lung completely with the intention of preventing any spread of the disease and allowing tuberculous cavities to heal. This was based upon the same principle of rest and immobilisation that underlay treatments for tuberculosis of the bones and joints where splints and plaster casts were employed to completely immobilise the affected area.^15^ Whilst the procedure had been shown to effect a marked improvement in the size of tuberculous cavities for some patients, it could be a very dangerous procedure with a risk of air embolisms, pleural shock, sepsis, emphysema and effusion.^16^ For patients undergoing this treatment, an artificial pneumothorax could be maintained for many months at a time, if not longer, by the regular insertion of air into the pleural cavity. Sometimes the treatment continued after discharge and patients would have to attend tuberculosis dispensaries for regular refills.

**Fig. 3.**
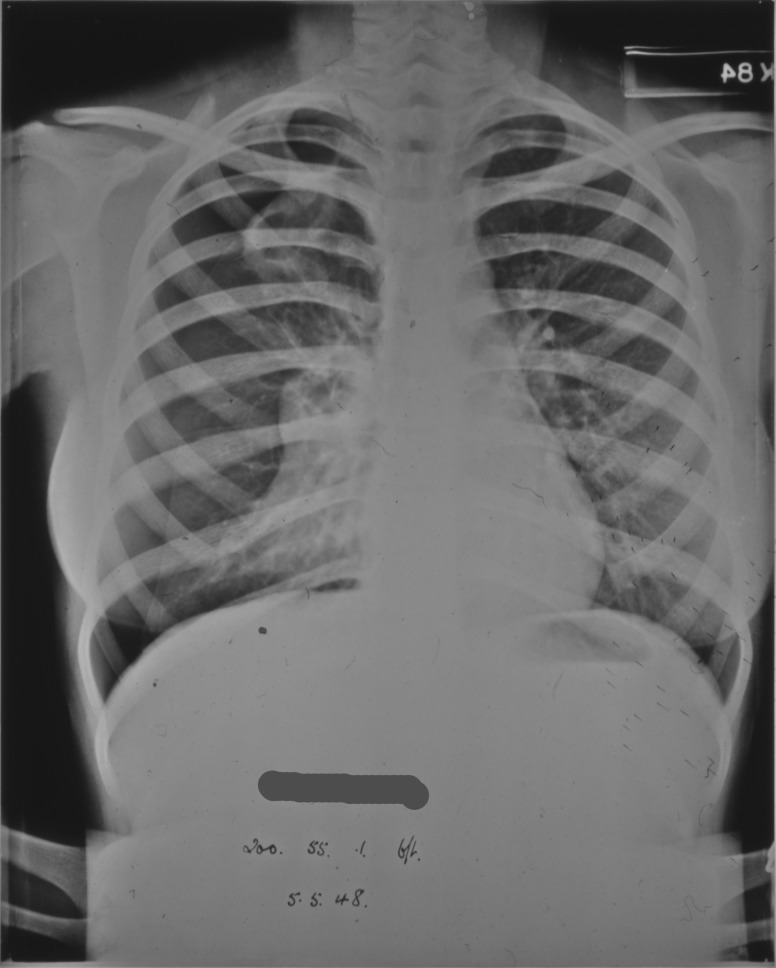
Early stages of artificial pneumothorax treatment evidenced by the area of opacity around the right lung. *Source*: Northumberland Archives: HOSP/STAN/7/1/2/1558_2.

**Fig. 4.**
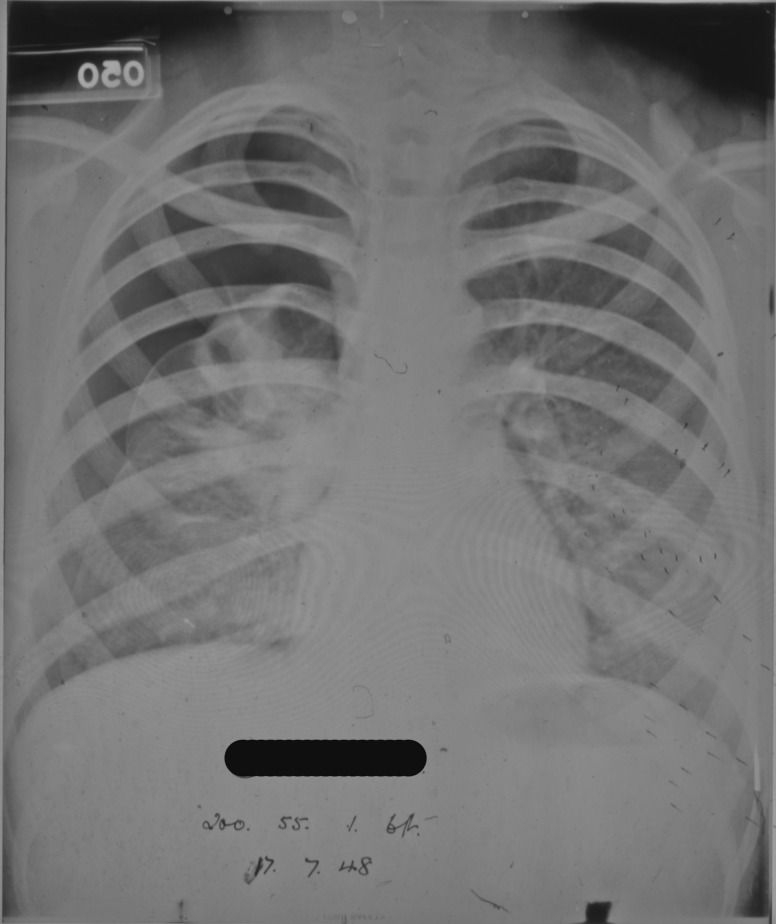
Progression of artificial pneumothorax treatment evidenced by an increase in the area of opacity round the right lung in comparison to Fig. 3. *Source*: Northumberland Archives: HOSP/STAN/7/1/2/1558_11.

In the case of patient 95/1947 no satisfactory collapse could be obtained due to fibrotic adhesions between the lung and the chest wall preventing the lung from collapsing. In some instances resection of the adhesions was undertaken surgically so that a proper collapse could be obtained. Radiographs from another patient show the initial attempts at a collapse evidenced in the opacity seen around the edge of the right lung in Figure 3, and the more advanced and successful collapse of the same lung in Figure 4, following resection of adhesions at the right apex.

With the failure of the artificial pneumothorax, patient 95/1947 was instead put forward for phrenic crush treatment, or phrenicotomy, and for an induced pneumoperitoneum. Again, these procedures were performed with the intention of resting the affected lung and were often resorted to when an artificial pneumothorax was not possible for whatever reason. By crushing the left phrenic nerve, situated in the neck, they would be able to disable the left diaphragm thus forcing the muscle to relax and lift up leading to a partial collapse and in turn resting of the lung. A pneumoperitoneum was often performed in conjunction with the phrenic crush and involved inserting air into the abdominal peritoneal cavity forcing the diaphragm up.^17^ For these procedures she had to be transferred to Shotley Bridge Hospital as the sanatorium did not have the facilities to carry them out. Unfortunately, upon completion of the procedures she was sent to a different sanatorium for monitoring and completion of treatment so we do not have full details of her subsequent progress. However, later correspondence in her file does confirm that she was eventually discharged in May 1950 and was presumably fit enough to leave sanatorium care.

The collection contains innumerable illustrations of surgical treatments such as this for a variety of different tuberculous conditions from the pulmonary procedures highlighted above to common methods in the treatment of tuberculosis of the bones and joints, such as arthrodesis. It is here that the radiographs become particularly useful in their illustrations and insights into the surgical procedures performed. Looking to a particular patient’s set of radiographs it is often possible to witness along the timeline of their treatment the manifestation and development of the disease, the effect of treatment or lack of it, and the subsequent level of success it had. In some instances there are up to 80 images for one patient.

## Access to the Collection

As part of a current project, each patient file and set of radiographs have been individually catalogued giving appropriate details to enable the selection of cases of interest to researchers. For example, each patient file is allocated its own unique reference number and the information recorded in the catalogue includes their dates of admission and discharge, original patient number, diagnosis, result of treatment, age on admission, sex, home town and the local authority responsible for the payment of their treatment. Where relevant any details of a child’s previous or later admissions are given and in all cases a list of associated records are also given to ensure that all sets of radiographs and case notes for each child can be easily located.

In addition all 14,660 radiographs have been digitised along with the early case notes. There was a change in the format of the case notes and the patient numbering system at the sanatorium in 1946 with the earlier notes being much larger in size. It is these larger records that have been digitised, comprising 949 individual files in total. An additional grant has been received by Northumberland Archives from the Wellcome Trust which will see the rest of the case files digitised in this way with completion planned for summer 2016.

Given the age of the Stannington records there are clear issues arising with regards to confidentiality and data protection as they are all less than 100 years old. However, the digitisation process has allowed us to redact any patient names from both the case notes and the radiographs thus making it possible for them to be accessed without the obstacle or worry of breaking patient confidentiality. All the digital copies of these records have been attached to and made available through Northumberland Archives’ online catalogue. Contact with several former patients throughout the project has meant that we have been able to gain their explicit support for the continued use of their records and in most instances the former patients we spoke to were particularly enthusiastic about the idea of their experiences still being relevant and able to inform future research. Access to physical copies of all the records can still be secured for purposes of academic research through the usual route of application through the NHS’s Caldicott Guardian.

The collection as a whole has much to offer in the field of medical history, amongst others, with the possibility existing to explore a range of different themes having only touched upon a handful here. Given the fact that it remained largely uncatalogued up until recently, it stands as a largely untapped resource accessible to researchers at every level and unique in the combination of its focus on children and the comprehensive nature of the run of patient records.

## Footnotes

^1^F. Miller *et al.*, *Tuberculosis in Children*, (London: J & A Churchill Ltd., 1963).

^2^M. Bernard, ‘Tuberculosis: A Demographic Analysis and Social Study of Admissions to a Children’s Sanatorium (1936–1954) in Stannington, Northumberland’ (unpublished PhD thesis, Durham University, 2003).

^3^Northumberland Archives: NRO 03897.

^4^The statistic for Northumberland is given as a percentage of new notifications of tuberculosis, both pulmonary and non-pulmonary, made that year, while that for the sanatoium is as a percentage of new admissions to the sanatorium that year.

^5^L. Bryder, *Below the Magic Mountain* (Oxford: Oxford University Press, 1988), 1.

^6^T. Waldron, *Paleopathology* (Cambridge: Cambridge University Press, 2009), 943.

^7^H. Altink, ‘From the Local to the Global: Fifty Years of Historical Research on Tuberculosis’, *Medical History*, 2015, 59, 3–5, 3.

^8^Public Health England, *Tuberculosis in the UK: 2013 Report* (2013) Available at: <http://webarchive.nationalarchives.gov.uk/20140714084352/http://www.hpa.org.uk/webc/HPAwebFile/HPAweb_C/1317139689583>, last accessed 19 January 2015.

^9^Northumberland Archives: HOSP/STAN/7/1/1/2745.

^10^T. C. Hunter, ‘A Case of Abdominal Tuberculosis with Extreme Emaciation’, *The Lancet*, 1928, 212, 752.

^11^R. A. Hobday, ‘Sunlight Therapy and Solar Architecture’, *Medical History*, 1997, 42, 455–72, 459.

^12^T. M. Allison, ‘Correspondence-Heliotherapy’, *British Medical Journal*, 1922, 2, 1281.

^13^T. C. Hunter, ‘Associations and Institutions: Stannington Sanatorium, Northumberland’, *The British Journal of Tuberculosis*, 1930, 24, 28–32, 30.

^14^Northumberland Archives: HOSP/STAN/7/1/1/1831.

^15^H. M. Davies, ‘A Consideration of the Treatment of Pulmonary Tuberculosis by Surgical Intervention’, *Tubercle*, 1920, 1, 209–19, 209.

^16^E. N. Packard, ‘Accidents During Operation’, in E. N. Packard, J. N. Hayes and S. F. Blanchet eds, *Artificial Pneumothorax* (London: Henry Kimpton, 1940), 85–95.

^17^B. K. Gupta, ‘Historical and Non-Pharmacological Management of Pulmonary Tuberculosis’, in S. K. Jindal, ed., *Textbook of Pulmonary and Critical Care Medicine Vol 2* (New Delhi: Jaypee Brothers Medical Publishers Ltd, 2011), 594–607, 598.

